# Heterogeneity of diagnosis and documentation of post-COVID conditions in primary care: A machine learning analysis

**DOI:** 10.1371/journal.pone.0324017

**Published:** 2025-05-16

**Authors:** Nathaniel Hendrix, Rishi V. Parikh, Madeline Taskier, Grace Walter, Ilia Rochlin, Sharon Saydah, Emilia H. Koumans, Oscar Rincón-Guevara, David H. Rehkopf, Robert L. Phillips

**Affiliations:** 1 Center for Professionalism and Value in Health Care, American Board of Family Medicine, Washington, District of Columbia, United States of America; 2 Department of Epidemiology and Population Health, Stanford School of Medicine, Palo Alto, California, United States of America; 3 Robert Graham Center, American Academy of Family Physicians, Washington, District of Columbia, United States of America; 4 Inform and Disseminate Division, Office of Public Health Data, Surveillance, and Technology, Centers for Disease Control and Prevention, Atlanta, Georgia, United States of America; 5 Coronavirus and Other Respiratory Viruses Division, National Center for Immunizations and Respiratory Disease, Centers for Disease Control and Prevention, Atlanta, Georgia, United States of America; University of Toronto, CANADA

## Abstract

**Background:**

Post-COVID conditions (PCC) have proven difficult to diagnose. In this retrospective observational study, we aimed to characterize the level of variation in PCC diagnoses observed across clinicians from a number of methodological angles and to determine whether natural language classifiers trained on clinical notes can reconcile differences in diagnostic definitions.

**Methods:**

We used data from 519 primary care clinics around the United States who were in the American Family Cohort registry between October 1, 2021 (when the ICD-10 code for PCC was activated) and November 1, 2023. There were 6,116 patients with a diagnostic code for PCC (U09.9), and 5,020 with diagnostic codes for both PCC and COVID-19. We explored these data using 4 different outcomes: 1) Time between COVID-19 and PCC diagnostic codes; 2) Count of patients with PCC diagnostic codes per clinician; 3) Patient-specific probability of PCC diagnostic code based on patient and clinician characteristics; and 4) Performance of a natural language classifier trained on notes from 5,000 patients annotated by two physicians to indicate probable PCC.

**Results:**

Of patients with diagnostic codes for PCC and COVID-19, 61.3% were diagnosed with PCC less than 12 weeks after initial recorded COVID-19. Clinicians in the top 1% of diagnostic propensity accounted for more than a third of all PCC diagnoses (35.8%). Comparing LASSO logistic regressions predicting documentation of PCC diagnosis, a log-likelihood test showed significantly better fit when clinician and practice site indicators were included (p < 0.0001). Inter-rater agreement between physician annotators on PCC diagnosis was moderate (Cohen’s kappa: 0.60), and performance of the natural language classifiers was marginal (best AUC: 0.724, 95% credible interval: 0.555–0.878).

**Conclusion:**

We found evidence of substantial disagreement between clinicians on diagnostic criteria for PCC. The variation in diagnostic rates across clinicians points to the possibilities of under- and over-diagnosis for patients.

## Introduction

Post-COVID conditions (PCC), sometimes referred to as “long COVID,” are a collection of health conditions that affect people for three or more months after infection with SARS-CoV-2 [1]. These conditions have been challenging to study, largely due to their status as a set of “potentially overlapping entities,” in the words of the US Department of Health & Human Services [2]. Understanding of PCC has evolved over time. Still, lack of detailed characterization creates difficulties for clinicians and researchers who try to treat and study the conditions [3]. The implementation of the diagnostic code for PCC (U09.9) in October 2021 gave researchers hope of a more standardized approach to diagnosing the condition.

Usage of the ICD-10 code, however, has differed substantially across clinicians, practices, and electronic health record (EHR) platforms, creating further complications for researchers. In a study from the United States Veterans Health Administration, researchers found that rates of PCC diagnosis following COVID-19 ranged from 3 to 41% across medical centers, largely due to differences in diagnostic practices [4]. Another study in the United States revealed that 35% of PCC diagnoses do not meet standards from the Centers for Disease Control and Prevention (CDC), and 60% do not meet World Health Organization (WHO) standards [5]. Meanwhile, a study from the United Kingdom found that users of different EHR platforms showed very different rates of documentation of PCC diagnosis [6].

The challenge created by inconsistent usage of the PCC ICD-10 code has created demand for alternative methods of identifying patients impacted by PCC. Researchers have largely tried to meet this demand through the development of machine learning-based strategies. Zhu, et al. (2023) used a small patient cohort and symptom surveys to train a classifier on clinical notes and achieved relatively good sensitivity, albeit with an expansive definition of PCC and an assumption of very high prevalence [7]. Rather than using clinical notes, other researchers have used tabular data to predict PCC. Pfaff, et al. (2022) used gradient-boosted decision trees with data contributed to the National COVID-19 Cohort Collaborative (N3C) to identify clinical and demographic features that were associated with specialty PCC clinic attendance [8]. Binka, et al. (2022) used ridge regression with administrative data from British Columbia for the same aim [9]. Among the high-prevalence test set of patients attending or diagnosed at a specialty PCC clinic, all achieved good performance, though their models’ generalizability to patients outside that setting is unknown.

These three machine learning based studies of PCC were designed to predict whether a given patient would be diagnosed at or eligible to attend a specialty PCC clinic. Our interest, however, was in evaluating the patterns of diagnosis that exist in the generalist primary care setting. To this end, we performed a series of descriptive and machine learning-based analyses that aimed to uncover the degree and potential sources of heterogeneity in the application of the ICD-10 code for PCC among clinicians in primary care, as well as potential commonalities among patients with PCC regardless of the presence of a diagnostic code.

## Methods

### Methods overview

We combined descriptive statistical analyses with machine learning to characterize the degree of diagnostic heterogeneity of PCC within primary care and to identify potential sources thereof. We first examined the distribution of PCC diagnoses across clinicians, which allowed for characterization of clinicians’ underlying propensity to diagnose PCC. Next, we analyzed the time between the first documentation of COVID-19 and the first documentation of PCC; this is an important component of guideline-concordant diagnosis. Then, to better understand the degree to which patient and clinician factors contributed to documentation of a PCC diagnostic code, we created a logistic regression with L1 regularization (i.e., LASSO regression) trained on the patients’ other diagnoses, demographic characteristics, practice, and clinician. Finally, we trained a natural language classifier on a sample of physician-annotated clinical notes to determine whether documentation may reveal common characteristics of patients with PCC, irrespective of the presence of the PCC ICD-10 code.

### Data source

**Fig 1:**
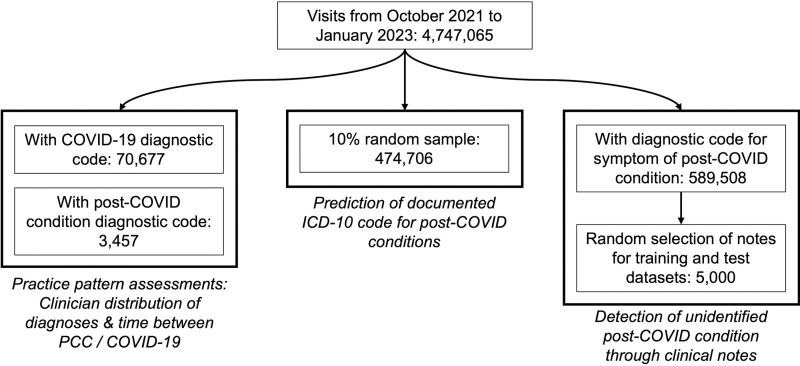
Data selection for the four included analyses. Samples were not mutually exclusive.

The American Family Cohort (AFC) is a collection of EHR data derived from a registry of mostly primary care clinics across the United States [10]. The records in AFC cover the healthcare encounters between over 12,000 clinicians and approximately 8 million unique patients. AFC began data collection on January 1, 2017, and continues to the period of this writing, although we only included data from up to November 1, 2023 (Fig 1). The patients were of diverse ages, races, ethnicities, and geographies. Approximately 20% of patients were missing data on race and ethnicity. For these patients, we used the highest probability race or ethnicity from a validated imputation based on name and census tract [11]. The final version of the data was accessed February 5, 2024. Data were not deidentified since we needed to use clinical notes.

### Assessment of practice patterns

We were interested in two proxies for understanding potential heterogeneity in clinicians’ diagnostic behaviors: first, the distribution of PCC diagnoses across clinicians; and second, the distribution of time between a patient’s first COVID-19 diagnosis and their first PCC diagnosis. Both of these analyses were conducted using the ICD-10 code U09.9 to identify patients who had been diagnosed with PCC between October 1, 2021, when the PCC ICD-10 code became available, and November 1, 2023 (Fig 1). All patients with a PCC diagnostic code were included in the descriptive analysis of how many PCC diagnoses each clinician recorded. COVID-19 was identified with ICD-10 code U07.1 or SNOMED code 840539006. Patients with both COVID-19 and PCC diagnostic codes were included in the analysis of time between recording the two diagnoses.

### Model of diagnosis code documentation

Following the method of Pfaff et al. [8], we developed a machine learning-based model to predict the documentation of an ICD-10 code for PCC (U09.9). As our primary interest was in examining diagnosis and documentation patterns in the context of general primary care, it was ideal that our dataset did not contain any records from specialty PCC clinics. We used as our analytic dataset a random 10% sample of visits between October 1, 2021 and November 1, 2023. This dataset included all ICD-10 codes recorded or retained within the patient’s problem list; patient demographics; date of visit; and both the practice and clinician visited. We collapsed all ICD-10 codes into the parent code: for example, the code F40.01 indicating agoraphobia would be collapsed to F40, which covers all phobic anxiety disorders. This resulted in a total of 1,596 parent codes. Because visits labeled with a PCC diagnostic code were vastly outnumbered by visits without, we used the Synthetic Minority Over-sampling Technique (SMOTE) to create synthetic data through interpolation of PCC-positives [12]. We next used a 70% split of the 10% sample to train a LASSO logistic regression model with the alpha hyperparameter optimized on a 10% split, then evaluated performance on the remaining 20%. Because our focus was on clinician- and practice-level heterogeneity around diagnosis, we reran this procedure while excluding these variables and tested comparative model fit with a log-likelihood test.

### Sample for natural language classifier

To create the sample of clinical notes to train the natural language classifier we selected patient visits between October 1, 2021 and January 9, 2023 based on the recorded reason for the clinical encounter. We included only notes from visits that had a SNOMED, ICD-9, or ICD-10 diagnostic code contained in the National Library of Medicine value sets “COVID-19 Potential Signs and Symptoms” (object identifier [OID]: 2.16.840.1.113762.1.4.1223.22) or one of the ten most reported symptoms of PCC in the cross-sectional survey of PCC patients conducted by Perlis, et al. [13] (Supplemental Material I). This method was designed to capture the largest number of potential PCC patients and may have missed patients who presented with uncommon symptoms.

Next, we included only patient notes having a length of at least 100 characters to eliminate most of the uninformative notes and extraneous data that could be mistakenly included in the clinical notes section (e.g., prescription refill requests or appointment scheduling logistics). We concatenated all notes for each unique combination of patient ID and visit date for the visits identified in the first step of the sampling process.

Finally, we randomly selected 5,000 clinical notes from unique patients among the subset of all notes meeting our criteria. Each note was from a single visit. We based this sample size on that of a similar study that achieved good model performance [14]. Many notes contained formatting marks, which we cleaned using regular expression-based functions. This was important not only for readability by the physicians who later labeled the documents, but also for avoiding training the NLP model on formatting marks – for example, identifying metadata or fonts from particular facilities.

### Natural language classifier development

Two family physicians (MT and GW) each reviewed 2,750 different notes. Both reviewed an overlapping set of 500 notes, which we used for calculating Cohen’s kappa for inter-rater reliability. The physicians used the criteria defined by the CDC to identify patients who were eligible for a PCC diagnosis [15]. Because they were using clinical notes from single visits and no structured data from patients’ charts, they also required attribution of the symptoms to a prior COVID-19 illness within the note. Within the overlapping subset, disagreements in classification were resolved by a third reviewer (NH).

We used three different NLP-based classifiers of increasing complexity: a tree ensemble model, a recurrent neural network (RNN), and a Transformer-based model. We optimized training hyperparameters on a 10% validation subset, fully trained each model on a 70% sample (i.e., 3,500 of 5,000 notes), and assigned the remainder to the test dataset.

For the first model, we trained an XGBoost model on two different numerical representations of the clinical notes: first, term frequency-inverse document frequency (TF-IDF) data – a form of regularized bag-of-words model [16,17] – and, second, as a document-level embedding generated from BioSimCSE, a model of biomedical text trained using contrastive learning [18]. XGBoost is a tree ensemble method that has proven competitive with deep learning methods on many types of sparse tabular data, including text [19].

For the second model, we employed a long short-term memory (LSTM) model [20,21]. Unlike bag-of-words approaches, which treat text as a static input with no consideration of word order or context, a LSTM RNN model can integrate the sequence of words and their dependencies into its predictions. We composed our model with an emmbedding layer, LSTM units, and a final dense layer, which we trained using binary cross-entropy loss and the AdamW optimizer.

The third model was a transformer pre-trained on deidentified clinical notes. Because it had shown superior performance on clinical classification tasks over general transformer models, we used Clinical-Longformer, which can accommodate texts of up to 4096 tokens (approximately equivalent to 3500 words) [22]. We first fine-tuned the Clinical-Longformer model on our corpus using the 70% training set and a masked language model objective to create a low rank adaptation (LoRA) adapter. Then, we optimized hyperparameters and trained the fine-tuned version of Clinical-Longformer to predict PCC from the notes.

Because only 1% of the notes related to PCC, we augmented presumptive PCC notes and under-sampled other notes. We first applied synthetic text augmentation to the positive cases using a WordNet-based synonym augmenter, which duplicates notes and randomly replaces words with synonyms, effectively increasing the variety of positive case samples [23]. We then employed random undersampling, in which a significant portion of the negative cases was randomly dropped, thereby reducing the disparity between positive and negative instances in the dataset. For the XGBoost model, which used tabular features, we used SMOTE instead of augmentation. With random sampling, we ensured that the ratio of negative to positive cases did not exceed 5:1.

We used the Optuna package in Python to optimize hyperparameters in all models [24]. In each case, we used evaluation loss on a 10% validation dataset as the cost function over 15 trials. Our primary outcome of interest was area under the receiver operating characteristic curve (AUC). For each model we also assessed positive/ negative predictive value at the optimal threshold (as determined by maximization of the F1 score within the validation dataset), Matthews correlation coefficient (MCC), F1 score on the negative class, and Brier score. We calculated confidence intervals for each model’s performance metrics by using a bootstrap with 1,000 repetitions.

### Software

Descriptive analyses were conducted in R, version 4.2, while modeling was conducted in Python 3.7. We used the xgboost package for prediction of PCC diagnostic code documentation [16]. We used the statsmodels package for LASSO logistic regression [25]; scikit-learn [26] and xgboost packages for the TF-IDF analyses; PyTorch for the RNN; and HuggingFace Transformers for the transformer [27]. (See Supplemental Material II for complete information on the computing and software environment.)

## Results

### Dataset characteristics

The AFC contained 9,722,653 visits conducted by 3,845 clinicians at 519 practices with 4,724,507 unique patients from October 1, 2021, to November 1, 2023. Among these, 116,659 patients had a diagnostic code for COVID-19 and 6,116 had a diagnostic code for PCC. A total of 5,020 individuals had diagnostic codes for both COVID-19 and PCC: 1,096 (18%) patients with a PCC diagnostic code did not have a diagnostic code for COVID-19.

### Patterns of documentation for the PCC diagnostic code

**Fig 2:**
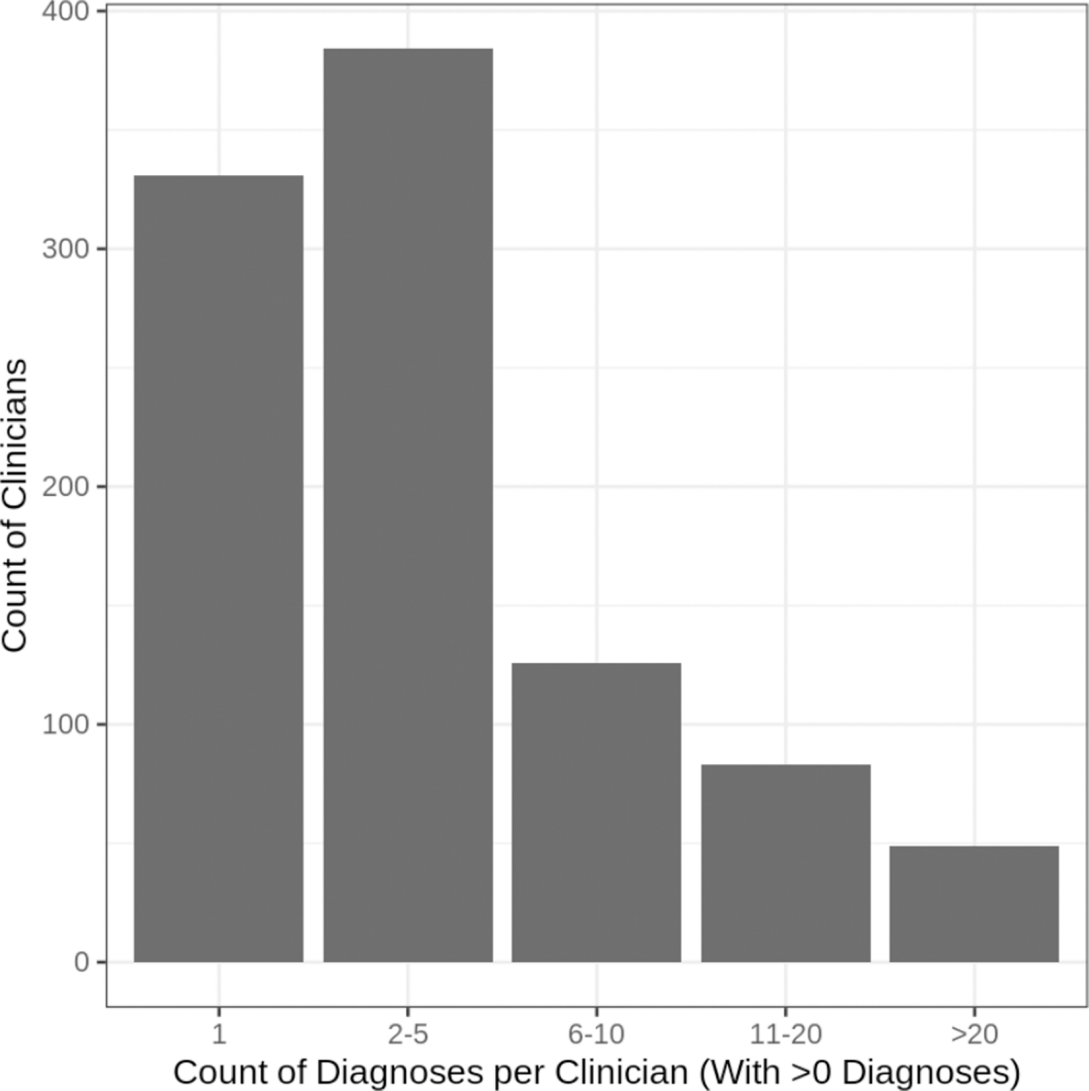
Diagnoses made for each of the 973 (out of 3,845 total) clinicians with at least one PCC diagnosis made from October 1, 2021 to November 1, 2023.

Of the 3,845 clinicians, 973 (25.3%) documented a PCC diagnostic code for at least one patient. The greatest number of PCC diagnoses took place in January and February of 2022, following the 2021 surge of infections driven by the emergence of the Omicron variant of SARS-CoV-2 (Supplemental Material III). The distribution of PCC diagnoses was highly right-skewed (Fig 2). A substantial share of the clinicians who diagnosed any PCC, 331 (34.0%), diagnosed only one patient with PCC. Six clinicians had over 100 patients with PCC, and the maximum number of PCC diagnoses for a single clinician was 224. These six clinicians, all of whom practice in different states without any public indication of working at a specialty PCC clinic, accounted for 15.6% (957 out of 6,116) of all PCC diagnoses documented with diagnostic codes; the clinicians with diagnostic propensities in the top 1% accounted for over a third of all PCC diagnoses (35.8%). Seven clinicians diagnosed more than 10% of their patients with PCC, and 35 diagnosed more than 5%.

### Time between COVID-19 and PCC diagnoses

Among patients with diagnostic codes for both COVID-19 and PCC, 295 (5.8%) had a PCC diagnosis recorded before their first documented diagnosis of COVID-19. An additional 3,078 (61.3%) individuals received their first PCC diagnosis less than 12 weeks after their first COVID-19 diagnosis, and 2,158 (43.0%) were diagnosed with COVID-19 and PCC less than 4 weeks apart. These thresholds represent the timing of diagnosis specified by the WHO and CDC, respectively. The mean time between first COVID-19 and first PCC diagnosis among those with both was 127 days, and the median time was 30 days.

### Predictive model of diagnostic code documentation

**Fig 3:**
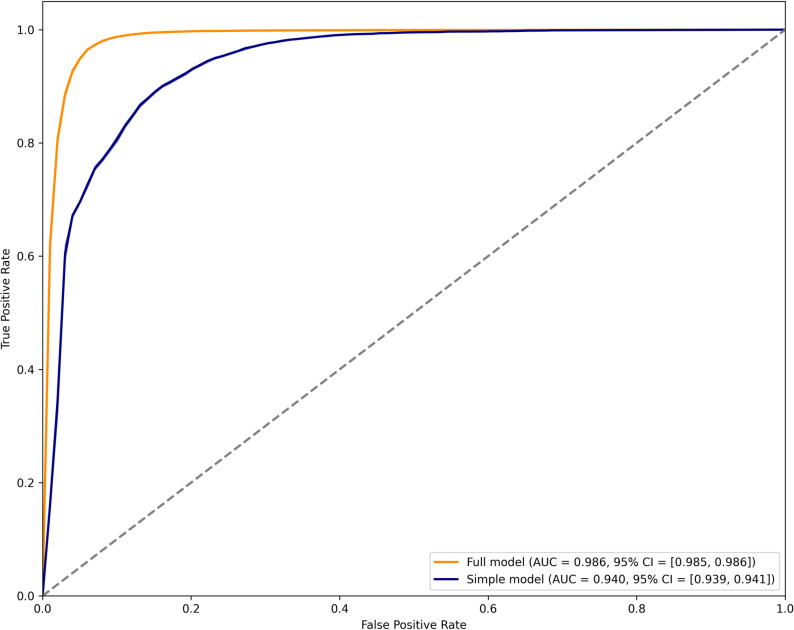
Receiver operating characteristic (ROC) curve for two LASSO logistic regression models of documentation of PCC diagnosis. The full model includes indicators for clinician and practice site, while the simple model excludes these.

The two LASSO logistic regression models trained on documentation of a diagnostic code for PCC achieved very good performance overall (see Supplemental Material IV for optimized hyperparameters for all models). The AUC for the full model was 0.986 (95% CI: 0.985–0.986) and was 0.940 (95% CI: 0.939–0.941) for the model excluding clinician and practice site indicators (Fig 3). Other statistics suggest that the model’s predictive ability significantly improved when clinician and practice site indicators were included (Supplemental Material V). A log-likelihood test showed that the full model was a significantly better fit for the data than the reduced model (p < 0.0001).

### Natural language classifier performance

In the annotated sample, 50 of the 5,000 notes were related to PCC. Interrater reliability calculations between the two physician reviewers showed a Cohen’s Kappa of 0.6 for PCC, which indicates moderate agreement beyond chance. The XGBoost model trained on TF-IDF features outperformed the model trained on embeddings (AUC: 0.793 versus 0.507).

The models had relatively low accuracy at identifying patients with PCC. The model AUCs were 0.757, 0.530, and 0.351 for the XGBoost, RNN, and transformer models, respectively (Table 1, Supplemental Material VI). Only the XGBoost model was better than chance when considering the AUC – a result echoed by the MCC metrics. Confusion matrices (Supplemental Material VII) showed that the RNN and transformer models achieved their best performance by classifying all individuals as not having PCC. Brier scores are good across all models, although this is likely attributable to the class imbalance and the predominance of negatives: calibration is good among negatives, but poor among positives. Calibration is best in the XGBoost model (Supplemental Material VIII).

**Table 1:** Performance characteristics of the three natural language classifiers (with 95% confidence intervals).

	AUC	F1 score (positive class)	F1 score (negative class)	Sensitivity	Specificity	MCC	Brier score
XGBoost	0.757 (0.585–0.905)	0.472 (0.200–0.696)	0.889 (0.825–0.940)	59.9% (25.0–88.9%)	85.2% (75.9–93.3%)	0.382 (0.078–0.645)	0.118 (0.060–0.118)
RNN	0.530 (0.321–0.733)	0.291 (0.080–0.516)	0.832 (0.748–0.902)	40.2% (9.1–75.0%)	78.4% (67.2–88.3%)	0.145 (-0.109–0.424)	0.142 (0.070–0.225)
Transformer	0.351 (0.206–0.506)	0.249 (0.100–0.394)	0.389 (0.250–0.537)	81.0% (54.6–100.0%)	25.1% (14.3–36.8%)	0.035 (-0.218–0.237)	0.142 (0.071–0.228)

Performance is based on physician review of notes within a 20% set-aside test set.

AUC, area under the curve; RNN, recurrent neural network.

## Discussion

Our results revealed substantial heterogeneity in the diagnostic behavior of clinicians in primary care in the 15 months following the introduction of the ICD-10 code for PCC. While a majority of clinicians in our dataset (75.7%) did not record a single diagnostic code for PCC, others applied the diagnosis very widely. Predictive models of PCC documentation showed that the inclusion of clinician and practice site identifiers significantly improved predictive ability, suggesting that these idiosyncratic factors, rather than differences in patient characteristics alone, may be key to understanding patterns of PCC diagnosis. Our data also showed that a majority (61.3%) of PCC diagnoses did not seem to meet the WHO criteria of at least twelve weeks between COVID-19 and PCC; nearly half (43.0%) did not meet the CDC definition of a minimum four weeks. We observed that the highest in PCC diagnoses occurred approximately four weeks after the January 2021 Omicron spike, while a secondary surge in PCC diagnoses followed the much smaller May 2021 wave of cases. Unlike the model of diagnostic code documentation, three natural language classifiers could not meaningfully identify PCC from the text of clinical notes. Of these models, the simplest performed best, although its performance was likely too poor to be useful in real world settings. The failure of these three models may indicate heterogeneous documentation patterns among clinicians, but it may also be a result of PCC’s highly variable clinical presentation.

PCC is a collection of symptoms that may commonly occur together but with wide variation. The CDC’s PCC symptom collection originally contained more than 1500 ICD-10 codes that may be indicative of PCC, which may mean that we have not sufficiently winnowed the most precise collection of symptoms to define the conditions. Clusters of patients may have different PCC clusters with little overlap between them, thereby producing variation in their presentation at primary care. In practice, primary care providers such as those represented in AFC have reported difficulties diagnosing and treating PCC patients in the face of often ambiguous definitions and standards [28]. The annotations of clinical notes from the two physicians demonstrated this issue: the level of agreement was only moderately above what would be expected by chance alone.

Our study revealed results that broadly accord with the findings of other studies. Prior research found low concordance between the ICD-10 code for PCC and the clinical criteria for the disease [5], and a right-skewed distribution of PCC diagnoses across clinicians [6]. Prior machine learning-based studies that focused on counterfactual probability of diagnosis had patients visited specialty PCC clinics all found different estimates of the mean risk of PCC following COVID-19, ranging from approximately 20% in Binka, et al. to over 40% in Pfaff, et al. [8,9]. This mirrors an even wider difference in estimates derived from prospective patient surveys, which range from 4.5% to 89%—a difference that has been attributed to heterogeneous case definitions [29].

Our results point to unmet needs for clinicians, patients, and researchers. Clinicians in general primary care settings have played an important role in identifying and managing PCC, yet report feeling poorly equipped to treat it holistically [28]. In practice, clinicians may choose to focus on individual symptoms rather than the collective condition and to treat PCC as a diagnosis of exclusion [30,31]. For this reason, Cau, et al. (2022) suggested that artificial intelligence could have a role in supporting PCC treatment across practices [32]. Meanwhile, patients face many unmet needs as their symptoms persist [33]. Researchers, too, face challenges in identifying patients with PCC in observational databases, making it difficult to characterize these conditions’ prevalence, trajectory, and treatment [34].

Our study had a number of limitations. First, the physician annotators only had access to notes from a single visit, and they did not have access to any diagnostic codes, vital signs, or labs. They were therefore limited in the amount of context they could use in their ascertainments, and their moderate inter-rater agreement likely placed a ceiling on model performance. A second limitation is that the Wordnet model that we used was not specialized for medical text. We are not aware of any clinically focused Wordnet dictionaries that we could have used in its place and did not find any gross errors on inspection. Next, the dataset was highly imbalanced, with PCC diagnoses included in a relatively small portion of visits. We took pains to appropriately augment the data, but are aware that its scarcity likely affected the performance of the trained models on the test sets. Finally, we cannot validate the dates documented for any diagnosis entered into an EHR. Thus, if a patient were diagnosed with COVID-19 in a setting not captured within our data (e.g., inpatient setting, emergency department), the accuracy of the date of first documented diagnosis recorded in the EHR depended upon the clinician who documented it. Similarly, the documented date of diagnosis may be delayed if the patient did not seek care upon initially testing positive outside of a clinical encounter. Thus, date associated with diagnosis code may not reflect when disease began and could be a “retrospective” code reflecting prior illness not captured previously in the EMR.

This study was strengthened by the use of the real-world data that captured the experience of diagnosing PCC in a diverse set of primary care settings. Our use of architectures validated on classifying COVID-19 diagnoses gave credence to our findings that even highly sophisticated NLP models struggle to accurately identify PCC patients from notes alone. Moderate inter-rater reliability metrics between the two physician annotators also highlighted the challenges of identifying PCC, even for expert reviewers.

Given the heterogeneity around diagnosis of PCC among the included clinicians, one area for future research may be the development of explicitly counterfactual approaches to identification of PCC cohorts. This may involve the development of clinician-specific models of diagnosis that indicate the likelihood that they would diagnose a patient with a given presentation. Researchers could then use a single diagnostic model across an entire population to standardize diagnosis across clinicians.

## Conclusion

In this study describing diagnostic and documentation patterns of PCC, we used a range of machine learning methods to characterize the role of clinician and practice site in predicting receipt of a PCC diagnosis and to try to identify PCC from clinical notes. We found extreme heterogeneity indicating that PCC diagnosis appears to be strongly related to factors other than health status. The impression of extreme heterogeneity in diagnosis was bolstered by the failure of three natural language classifiers to meaningfully detect PCC in clinical notes. Lack of clear diagnostic criteria with applicability in primary care may contribute to this simultaneous under- and over-diagnosis, which creates barriers to effective care with for patients with PCC.

## Supporting information

Supplemental Material I Symptoms included in identification of candidate cohort. Object identifiers are from the National Library of Medicine Value Set Authority Center.
Supplemental Material II
Details of computing environment.
Supplemental Material III
Monthly diagnoses of PCC in the time since the PCC ICD-10 code became available October 2021 – October 2023.
Supplemental Material IV
Optimized hyperparameters.
Supplemental Material V
Detailed statistics (with 95% confidence intervals) for predictive models of PCC documentation.
Supplemental Material VI
Receiver operating characteristic curve for the three natural language classifiers trained to identify PCC. AUC = area under the curve; CI: credible interval; RNN = recurrent neural network. 95% credible intervals are the result of 1,000 bootstrap samples of the test dataset with model predictions.
Supplemental Material VII
Confusion matrices for the three NLP models.
Supplemental Material VIII
Calibration plot of the three models. (PDF)
