# Supplementary material for: Heterogeneity of diagnosis and documentation of post-COVID conditions in primary care: A machine learning analysis
Source: PLoS One. 2025 May 16;20(5):e0324017. doi: 10.1371/journal.pone.0324017 (PMC12083802; doi:10.1371/journal.pone.0324017)

Supplemental Material for "Heterogeneity of Diagnosis and Documentation of Post COVID Conditions in Primary Care: A Machine Learning Analysis"

I. Symptoms included in identification of candidate cohort. Object identifiers are from the National Library of Medicine Value Set Authority Center.

**Symptom**	**Object Identifier(s)**
Shortness of breath	2.16.840.1.113762.1.4.1182.47
Fatigue	2.16.840.1.113762.1.4.1146.862 and 2.16.840.1.113762.1.4.1146.860
Headache	2.16.840.1.113883.17.4077.3.1027
Loss of smell	2.16.840.1.113762.1.4.1146.1202 and 2.16.840.1.113762.1.4.1146.1201
Brain fog	2.16.840.1.113762.1.4.1222.1377
Poor memory	2.16.840.1.113883.3.3616.200.110.102.3221 and 2.16.840.1.113883.3.3616.200.110.102.6310
Dizziness	2.16.840.1.113883.3.3616.200.110.102.3223 and 2.16.840.1.113883.3.3616.200.110.102.6315
Depressed mood	2.16.840.1.113883.3.600.145
Anxious mood	2.16.840.1.113762.1.4.1021.94
Sleep disruption	2.16.840.1.113762.1.4.1222.1318

II. Details of computing environment

All computations were run on a server hosted on the premises of Stanford University that is designed to host high-risk data. The server runs Ubuntu Linux 20.04 and compute nodes used for this project were provisioned with 8 CPUs, 1 NVIDIA A100 GPU, and 124 GB memory. Data wrangling was performed in R 4.2 using tidyverse and data.table packages. Other analyses were conducted in Python 3.11.10 with the following dependencies: "altair>=5.5.0", "datasets>=3.3.2", "fastparquet>=2024.11.0", "ipykernel>=6.29.5", "joblib>=1.4.2", "jupyterlab>=4.3.5", "matplotlib>=3.10.1", "numpy>=2.2.3", "optuna>=4.2.1", "pandas>=2.2.3", "peft>=0.14.0", "scikit-learn>=1.6.1", "seaborn>=0.13.2", "sentence-transformers>=3.4.1", "statsmodels>=0.14.4", "torch>=2.6.0", and "xgboost>=2.1.4".

III. Monthly diagnoses of PCC in the time since the PCC ICD-10 code became available October 2021 – October 2023.

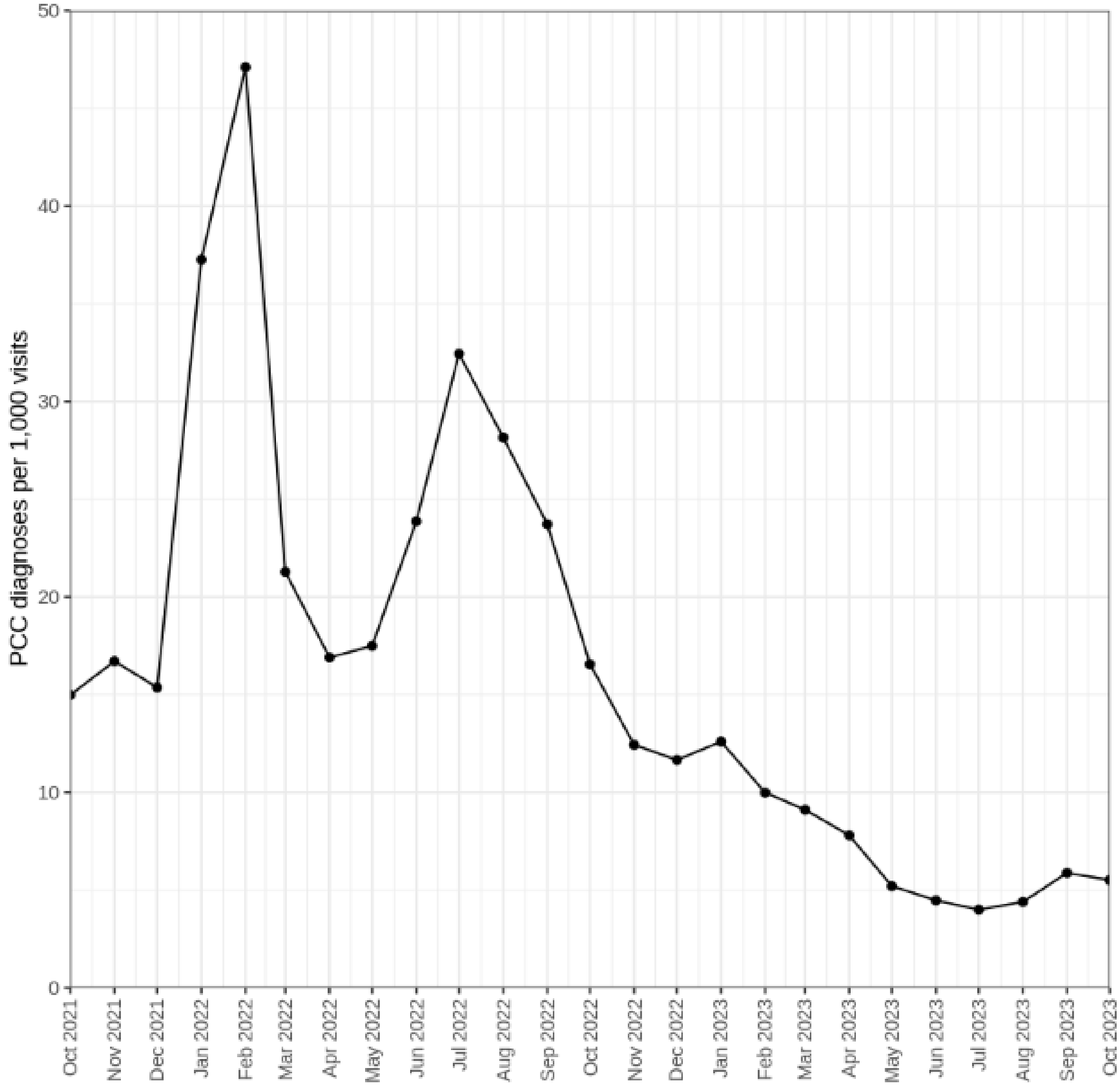

IV. Optimized hyperparameters

Included hyperparameters were optimized on a 10% validation set for each model using the Optuna package.

Models of Diagnostic Code Documentation

1. Full model
    Alpha: $4.9207 * 10^{-4}$

2. Simple model:
    Alpha: $2.7925 * 10^{-4}$

Classification Models from Clinical Notes

1. XGBoost (TF-IDF)
    Maximum depth: 10
    Learning rate: 0.1441
    Number of estimators: 448
    Minimum child weight: 5
    Gamma: $3.3687 * 10^{-6}$
    Subsample: 0.6645

2. RNN
    Embedding dimensions: 164
    Hidden dimensions: 207
    Number of layers: 2
    Dropout: 0.4973
    Learning rate: $1.6351 * 10^{-3}$

3. Transformer (finetuned Clinical-Longformer)
    Learning rate: $2.0015 * 10^{-5}$
    Weight decay: 0.0131
    LoRA rank: 17
    LoRA alpha: 30
    LoRA dropout: 0.1817

V. *Detailed statistics (with 95% confidence intervals) for predictive models of PCC documentation*

	Full model	Simple model
Optimal threshold (maximum F1 score on positive class)	0.550	0.550
Matthews correlation coefficient	0.905 (0.904-0.907)	0.741 (0.739-0.743)
F1, positive class	0.953 (0.953-0.954)	0.873 (0.872-0.875)
F1, negative class	0.952 (0.951-0.952)	0.866 (0.865-0.867)
Brier score (note: lower score indicates better performance)	0.038 (0.037-0.038)	0.095 (0.094-0.096)

VI. Receiver operating characteristic curve for the three natural language classifiers trained to identify PCC. AUC = area under the curve; CI: credible interval; RNN = recurrent neural network. 95% credible intervals are the result of 1,000 bootstrap samples of the test dataset with model predictions.

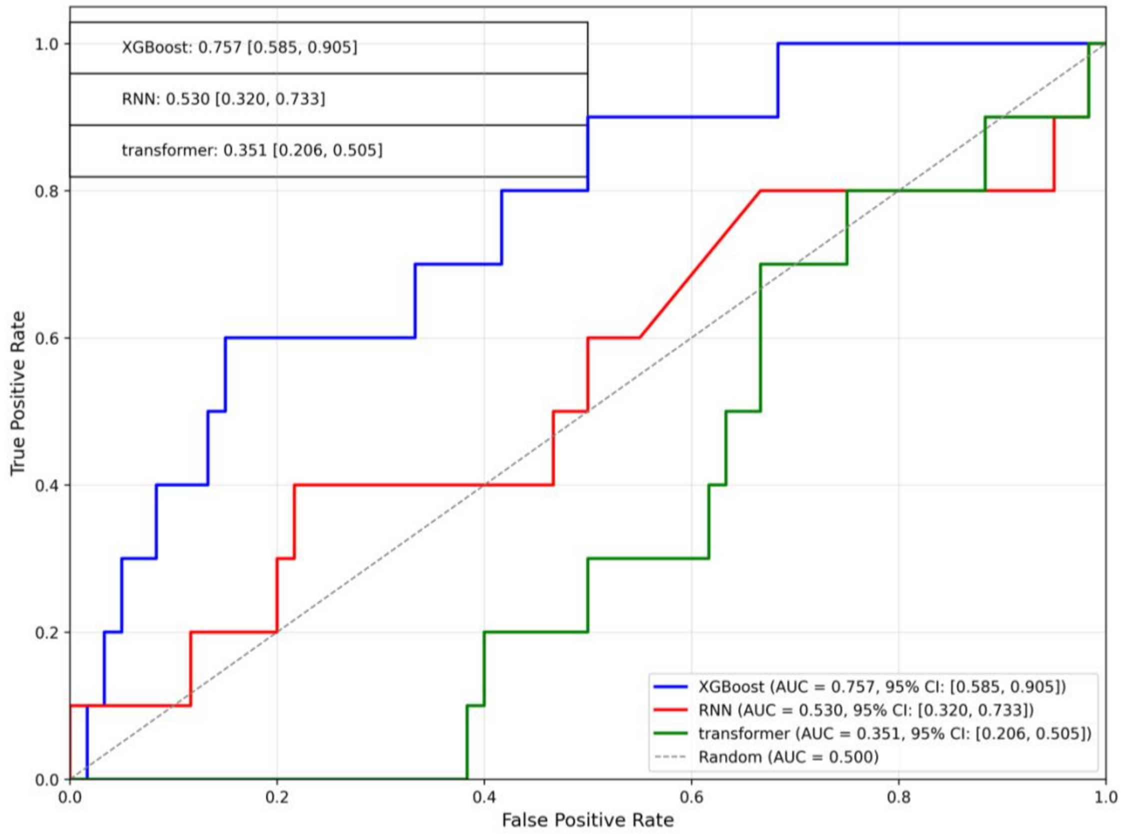

VII. Confusion matrices for the three NLP models

XGBoost

True \ Predicted	0	1
0	51	9
1	4	6

RNN

True \ Predicted	0	1
0	47	13
1	6	4

Transformer

True \ Predicted	0	1
0	15	45
1	2	8

VIII. Calibration plot of the three models

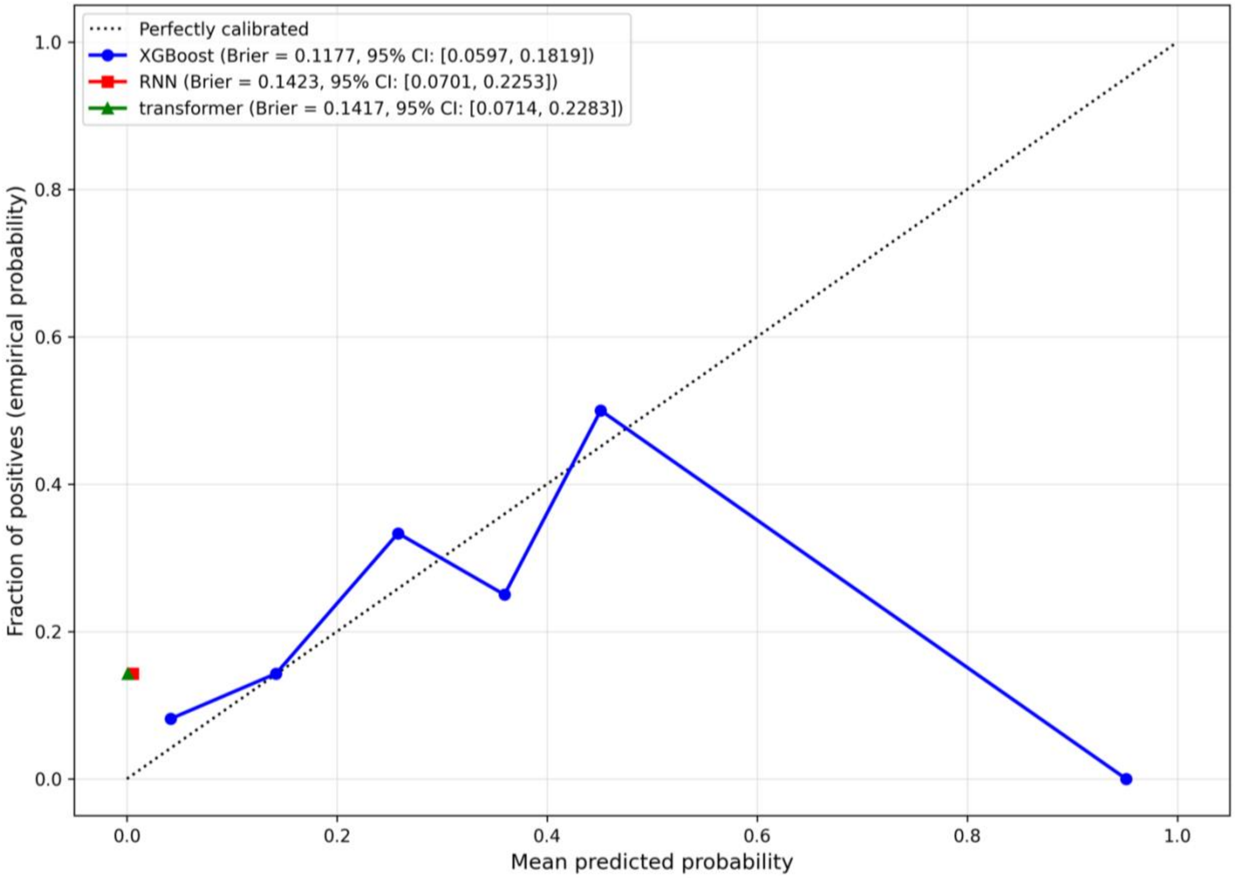

Supplement: Supplemental Material I — Symptoms included in identification of candidate cohort. Object identifiers are from the National Library of Medicine Value Set Authority Center. Supplemental Material II Details of computing environment. Supplemental Material III Monthly diagnoses of PCC in the time since the PCC ICD-10 code became available October 2021 – October 2023. Supplemental Material IV Optimized hyperparameters. Supplemental Material V Detailed statistics (with 95% confidence intervals) for predictive models of PCC documentation. Supplemental Material VI Receiver operating characteristic curve for the three natural language classifiers trained to identify PCC. AUC = area under the curve; CI: credible interval; RNN = recurrent neural network. 95% credible intervals are the result of 1,000 bootstrap samples of the test dataset with model predictions. Supplemental Material VII Confusion matrices for the three NLP models. Supplemental Material VIII Calibration plot of the three models. [file pone.0324017.s001.pdf]
